# Supplementary material for: Adsorption of Methylene Blue and Tetracycline by Zeolites Immobilized on a PBAT Electrospun Membrane
Source: Molecules. 2022 Dec 22;28(1):81. doi: 10.3390/molecules28010081 (PMC9822180; doi:10.3390/molecules28010081)
Supplement: Supplementary file 1 [file molecules-28-00081-s001.zip › molecules-2100565-supplementary.pdf]

Supplementary information: *Adsorption of methylene blue and tetracycline by zeolites immobilized on a PBAT electrospun membrane*

David Picón[a,b], Alicia Vergara-Rubio[a,c], Santiago Estevez-Areco[d], Silvina Cerveny [e,f], Silvia Goyanes[a,b]*

[a] Universidad de Buenos Aires. Facultad de Ciencias Exactas y Naturales, Departamento de Física, Laboratorio de Polímeros y Materiales Compuestos (LP&MC). Ciudad Universitaria (C1428EGA), Ciudad Autónoma de Buenos Aires, Argentina.

[b] CONICET - Universidad de Buenos Aires. Instituto de Física de Buenos Aires (IFIBA). Ciudad Universitaria (C1428EGA), Ciudad Autónoma de Buenos Aires, Argentina.

[c] IIIA-UNSAM-CONICET, Instituto de Investigación e Ingeniería Ambiental, Escuela de Hábitat y Sostenibilidad, San Martín, Provincia de Buenos Aires B1650, Argentina;

[d] Instituto de Física de Materiales Tandil (UNCPBA) and CIFICEN (UNCPBA-CICPBA-CONICET). Pinto 399 (B7000GHG), Tandil, Buenos Aires, Argentina.

[e] Centro de Física de Materiales (CSIC, UPV/EHU)-Materials Physics Center (MPC), Paseo Manuel de Lardizabal 5,San Sebastián 20018, Spain.

[f] Donostia International Physics Center (DIPC), San Sebastián 20018, Spain.

*Corresponding autor (e-mail address: goyanes@df.uba.ar)

1. Methods

1.2 Characterizations

1.2.1 Infrared spectroscopy

Infrared spectra were obtained using a Fourier Transform Infrared Spectrometer (Jasco FT-IR 4100, Japan) equipped with an attenuated total reflectance module (ZnSe crystal). Spectra were recorded in a range from 4000 to 600 cm^{-1}, at a resolution of 4 cm^{-1} averaging 24 scans per sample. Spectra were normalized with respect to the band at 1710 cm^{-1}, which is expected to not change after acid treatment.

1.2.2 Thermal characterization

Thermal properties were studied by thermogravimetric analysis (TGA, Shimadzu, Japan). Samples of each membrane were placed in aluminium pans in the TGA-DTA balance. Tests were performed under nitrogen atmosphere (flow rate of 30 mL/min) from 30 to 450 °C at a heat rate of 10 °C/min.

2. Results

2.1 Infrared spectroscopy

Figure S1 presents the FTIR spectra of PBAT membranes after/before acid treatment with HCl 6 M. Both spectra show the characteristic bands of PBAT. No significant difference are observed, which indicates that the polymer did not undergo chemical modifications as consequence of the acid treatment.

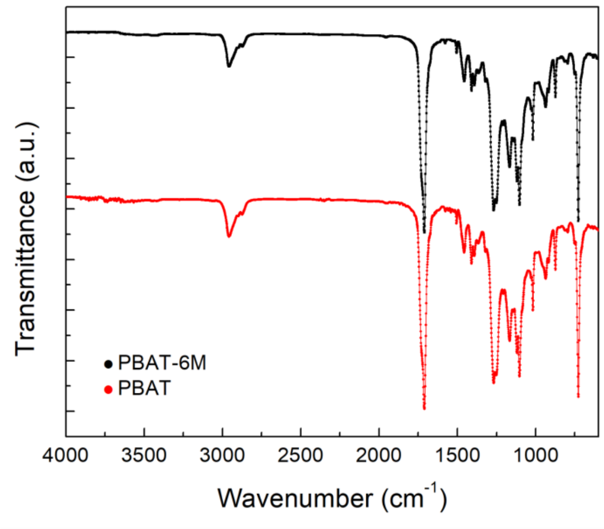

Figure S1. FTIR spectra of PBAT and PBAT-6M.

2.2 Thermal analysis

Figure S2 compares the TGA curves of a PBAT membrane with those corresponding to PBAT acid-treated membrane (with/without zeolites). Thermal degradation of electrospun membrane occurs in a single step from 330 °C to 440°C, leaving a residual mass of ~7% at 500 °C. There were no differences between the degradation of untreated and acid-treated PBAT membranes, so that thermal stability of polymer was preserved after the zeolites functionalization. The incorporation of zeolites within the PBAT fibers did not alter thermal degradation of the membrane either.

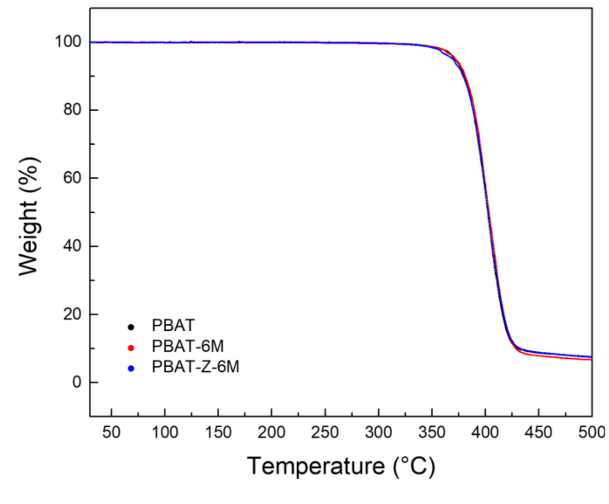

Figure S2. Thermal degradation of PBAT-based membranes.
